# Grouping Together to Fight Cancer: The Role of WeChat Groups on the Social Support and Self-Efficacy

**DOI:** 10.3389/fpubh.2022.792699

**Published:** 2022-03-08

**Authors:** Fangqi Zhong, Li Pengpeng, Zhuo Qianru

**Affiliations:** College of Communication, National Chengchi University, Taipei, Taiwan

**Keywords:** self-efficacy, disease self-management, WeChat groups, cancer mutual aid, social support

## Abstract

With the increasing number of cancer survivors, the question of how to coexist with cancer has become more and more pressing. This research uses a mutual help WeChat group organized by cancer patients as the research field to observe the daily interactions of cancer patients, so as to improve understanding of how social media technology can help cancer patients in the treatment and recovery process. The study found that the WeChat group is the main source of health knowledge for the participating cancer patients, and that when compared to traditional web-based patient mutual aid communities, the WeChat group is a more timely, popular, continuous, and accurate source of information. Patients in the group can listen and respond to each other's questions and worries, providing both an outlet for patients to vent their emotions and concerns and a source of recognition and encouragement. In addition, this study found that the WeChat mutual aid group improves patients' self-efficacy of disease on four levels: successful experience in curing patients, imitating patients' behavior, verbal persuasion, and emotional support.

## Introduction

In 2020, nearly 20 million people worldwide were diagnosed with cancer and it is estimated that this number will surpass 70 million in 2050 (1). Proportionally, an estimated 41.24% of all people born today will be diagnosed with some type of cancer during their lifetime (2). Fortunately, patient prognosis and overall survival rates have improved dramatically due to advances in early cancer diagnosis and treatment techniques (3). From a medical perspective, the number of cancer survivors is increasing, and many cancers have progressed from being acute to chronic conditions. How patients should cope with the physical and psychological trauma they experience during treatment and recovery and how they can improve their long-term quality of life are issues that need to be addressed in modern care.

Cancer and its treatment can induce a range of serious biological/psychological side effects that can have a long-term impact on patients' daily lives. Usually, after a series of treatments such as chemotherapy, radiotherapy, and surgery, the quality of life of cancer survivors is visibly impaired and many suffer from side effects and after-effects. Likewise, cancer patients are prone to anxiety and depression due to the long duration of treatment, the variability of the disease, and the number of complications (4). For example, about 80% of cancer patients suffer from cancer-related fatigue [CRF; (5)], which is a persistent feeling of uncontrollable exhaustion after treatment; this subjective fatigue does not resolve with rest. Therefore, optimizing the care of cancer patients is of great relevance for their adjustment to chronic physical discomfort and its psychological impact (6).

Traditionally, patients have received information about their illnesses primarily through verbal communication from their primary care physicians. This form of doctor-patient communication has long been hampered by time constraints, inadequate health literacy, and communication gaps (7). Unfortunately, at least in China, with the increasing number of cancer patients, traditional oncology services have limited ability to provide resources such as time and psychological support to patients (8), and their care systems can no longer meet patient needs. In recent years, with the development of media technology, various online or digital health interventions have been gradually applied in the healthcare field. Based on the spiritual care of patients, many hospitals have developed various supplementary applications to provide cancer patients with broad and promising platforms for health information and communication. For example, some hospitals in China have been able to enhance communication between patients and nurses with standardized health education through the WeChat public platform, answering patients' questions on time and overcoming the drawbacks of traditional verbal education (9). This authoritative, expert-led, top-down online health system overcomes the time, space, and equipment constraints of medical outpatient clinics and is easy to implement, saving financial and material resources (11) so that patients can feel the constant support of their doctors (10).

However, it has also been shown that this top-down passive, traditional format of health education has limited impact on behavioral change, although it may improve patients' knowledge and skills (12). However, changes in disease self-management behaviors are not negligible for cancer patients because they not only help to regulate and control the treatment process but also reduce the chance of disease recurrence (13). Here, the concept of self-efficacy can be helpful. The concept of self-efficacy focuses on the patient and is widely used to examine the patient's adjustment to and treatment of his or her illness and to predict his or her health behaviors (14). The concept suggests that a patient's positive belief that they can beat the disease has a positive impact on the development of health behaviors (15). That is, if patients' sense of efficacy increases, they can consciously engage in self-management behaviors such as eating right, exercising appropriately, and taking medication as prescribed (16). This is helpful for patients to return to society and normal life and stabilizes their psychological state and reduces their relapse rate later in life (17). In addition, since the medical community has not yet established a definite theory of cancer pathogenesis, this also provides room for the kinetic role of psychiatric efficacy to play a role. Therefore, exploring patients' behavioral changes due to their beliefs from the perspective of self-efficacy compensates for the difficulty of passive health education to stimulate patients to perform rehabilitation behaviors.

In the light of the above, living with cancer has become an increasingly important trend in human society and there is an urgent need to address the long-term physical and psychological damage caused by cancer. Although initial evidence suggests that cancer patients are benefiting from new media and internet technology, the ratio of doctors to patients is not as high as it should be. Due to this disparity in the doctor-patient ratio and the need for patient self-management behaviors, efforts to improve cancer patient support models are continuously being made and evaluated (1). Currently, there is a lack of research in the Chinese academic community on how patients can help themselves and each other in the absence of hospitals, healthcare providers, and authoritative experts. What is the role of chat rooms, which are now mainstream mobile terminal applications? Therefore, this study uses a mutual help WeChat group organized by cancer patients^1^ as a research field to observe the daily interactive experiences of cancer patients in China. It also explores the social logic and appearance of the WeChat group to improve patients' self-management behaviors by enhancing patients' sense of efficacy, so as to supplement the research experience of cancer patients' self-help/mutual assistance network practice.

## Literature Review

### Doctor-Patient and Patient-Patient Communication in the China

In the past, nurses and bedside nurses provided care to patients according to the requirements of the hospital. Although they were able to meet the requirements of the hospitalization period, the content of nursing care was uniform, its duration was short, and the actual condition of patients was not taken into account, resulting in poor quality of care (9). With the new service concept of humanistic care proposed in modern nursing, Chinese hospitals have shifted to a person-centered approach in the practice of patient care, combining professional nursing skills with humanistic factors (18). Academics have also summarized models such as the patient-centered (19), personalized care (20), and collaborative care model (21) to provide targeted care according to treatment requirements and patient needs to help patients achieve the desired outcomes and ultimately improve their wellbeing. In general, the improvement in traditional medical care is based on the following aspects (22): (1) establishing a timely communication channel between doctors and patients and formulating care plans with patients and their families; (2) conducting health education, distributing informational pamphlets, and explaining relevant knowledge; (3) emphasizing psychological interventions, eliminating the negative emotions of patients, and helping them build confidence in treatment; (4) patients are instructed to follow healthy eating, sleep and exercise patterns to improve their long-term lifestyle.

The conventional approaches are constantly updating their methods of care and aiming to provide more comprehensive services to patients. However, although it is still at the small-scale trial stage, the personalized care approach cannot avoid the need for more healthcare staff and training procedures. Given the disparity in the current doctor-patient ratio in China, the conditions are not sufficient to promote and implement such a model of care. To solve this dilemma, traditional hospitals have also relied on media technology to extend new means of supplementary care provision to patients through media platforms. For example, hospitals have posted QR codes in wards to disseminate relevant knowledge through the WeChat platform on topics such as illness, rest, psychology, rehabilitation, reminders for follow-up consultations, and post-operative medication (9). For example, daily care interventions for post-discharge laryngeal cancer patients through nurse-patient WeChat groups are shown to be beneficial for their post-operative recovery, social functioning, emotional functions, and mental health (23). The hospital-led Interactive Health Communication App (IHCA) is also considered to have overcome the weaknesses of traditional doctor-patient communication with its 24-h availability, interactivity, and multimedia capabilities, which can strongly enhance the scope of hospital services, reach more patients, and provide customized health information (24). It is evident that all such media technology platforms provide online access platforms for patients to reinforce and consolidate disease-related knowledge, answer difficult questions on time, and give correct guidance (11).

In summary, although studies in China have confirmed that various social media channels provide useful new platforms for hospital-patient communication, there is a lack of research exploring social media functions in the context of patient-patient interactions. In China, online technology has progressed to the point of intelligent interconnection (25), and applications such as WeChat, QQ, and Weibo are representative media technology products that are popular, portable, and easy to use, facilitating daily communication and information-sharing for a wide range of users (26). According to official reports, the number of active WeChat accounts has exceeded 1.1 billion. WeChat is a Chinese multifunctional instant messaging, social media and mobile payment application. It is described as China's “universal application” because it has a wide range of functions, such as text messaging, call voice messages, broadcast messages, video conferencing, video games, photo and video sharing, and location sharing. Among them, the WeChat group function has become the most active conduit for online social interaction through mobile (27). Numerous studies have demonstrated that participation in WeChat groups has positive effects on members' willingness to share knowledge (28), information dissemination (29), pleasure of helping others (30), and emotional ties (31).

In addition, although some attention has been paid to the experience of patient-patient interactions on social media in Europe and the United States, the research has focused on public web pages or media websites. Compared to Weibo or BBS groups, these are characterized by a large number of members, anonymity, weak links, low self-exposure, slow replies, non-portability, and high health/media literacy requirements (32–35). Compared to web-based public social platforms, WeChat as an app on smartphones for conversation (like WhatsApp and Line), its groups are more like a highly private social extension of private chat in terms of functional attributes and communication methods and processes, focusing more on interpersonal interactions and building relationships through two-way conversations (36). Given the timely, private, and close nature of WeChat, it is reasonable to believe that the experience of web-based patient support communities in Europe and the United States may not be fully applicable to the Chinese patient support field where WeChat is the main vehicle. Therefore, this study uses WeChat groups, the most popular and frequently used social media application in China, to explore how cancer patients use the WeChat group function for daily interaction and mutual support, in order to further determine how this new social media technology can help cancer patients in their treatment and recovery process.

### Health Literacy and Social Support

Seeking social support is an important mechanism for coping with cancer. Both instrumental (seeking support for advice, assistance, or information) and emotional social support seeking (seeking moral support, sympathy, and understanding) as important behaviors relevant to problem-focused coping (37). Emotional social support can help with the processing of personal concerns and psychological distress, while instrumental social support seeking can result in tangible and information-based support (38).

Health literacy is the ability of an individual to obtain, process, and understand basic, necessary health information and apply this information to receive treatment or make health-related decisions (39). Nowadays, the health literacy of the population has become a global concern (40). Research in the health field has demonstrated that low health literacy makes it difficult for people to understand disease prevention measures, screen medical information, and make treatment-related decisions, which can be detrimental to treatment and subsequent recovery (41). For this reason, population health literacy is increasingly recognized as a key factor in the overall cancer care landscape, and effective communication regarding cancer is considered a top clinical and public health priority (42). Currently, most empirical studies on health literacy come from European and American countries. They found that even in developed countries, more than 60% of adults cannot access health information and make appropriate medical decisions on their own (43). In particular, when patients leave the doctor's office, they retain only 50% of the critical information provided by the doctor and often complain that the doctor does not explain their illness in terms that they can understand (39). Thus, the prevalence of low health literacy means that most patients have difficulties with verbal and written expression, which limits their understanding and communication of cancer screening and symptoms and adversely affects both diagnosis and self-care (44).

Although a wealth of information on cancer and its care is available to patients through web browsing, it places demands on their media and health literacy. Specifically, the information available on the web may be too dense and unreadable for patients without medical knowledge and requires them to be proficient in actions such as locating and searching sites, spelling, navigating content, and linking to other sites (43). Otherwise, web browsing results in hours of futile and frustrating searching (39). Fortunately, these shortcomings are compensated for by mutual help WeChat groups organized by cancer patients on social media. In particular, the low cost and popularity of smartphones provide patients with access to terminal interaction anywhere and anytime. Mobile-based social media software has a low entry threshold of knowledge making it easily accessible to people with low literacy and health literacy (45); even illiterate patients can communicate with each other through voice messaging. In the context of this new medium, patients can build intimate relationships as partners without the constraints of time, space, and fixed devices (46). They also have more opportunities to discuss common experiences, health problems, and treatments with other patients (45), giving them greater confidence in their coping skills, which in turn facilitates health literacy (47).

While the above concerns about health literacy are instrumental support, social support, and emotional social support regarding mental health cannot be ignored. Patients with cancer often have to cope with a deteriorating physical condition and a range of treatments with strong side effects. Even after treatment completion, concerns about the after-effects of the treatment and the recurrence of the tumor remain. Many cancer patients develop psychiatric complications due to the physical and mental exhaustion accompanying treatment, and nearly 60% of cancer patients even experience depression during treatment (48). To preserve their mental health, cancer patients often require above-average levels of interpersonal and emotional support (49). This support comprises moral or material help and support from various sectors of society, including parents, relatives, friends, and social groups (50). The receipt of more support from their surroundings effectively alleviates patients' negative emotions and has a positive effect on their motivation to participate in treatment and their desired disease outcome (51). Due to the positive effect of emotional support on patients, clinical empathy has become popular in healthcare practice (19). This means that healthcare professionals are committed to understanding patients' experiences, needs, and thoughts and communicating with them accurately to optimize treatment outcomes (52). Medical and patient empathy are recognized to help patients develop the confidence and courage to cope with their disease, thereby improving the quality of care (53). However, cancer centers and treatment facilities often do not provide enough of this emotional labor due to staff and funding shortages (54).

The patient support model may be able to compensate for the lack of clinical emotional support. The core of the patient support model is to promote the establishment of healthy behaviors through patient support teams and monitor each other to achieve good long-term lifestyle habits (55). On the one hand, patients can eliminate anxiety and depression from their mental experiences; on the other hand, they can acquire correct recovery methods, such as diet and exercise, from their experiences. In the past, patients mostly got acquainted in the queues for consultation or treatment and in the setting of inpatient wards, which are characterized by low scope, distant relationships, and weak continuity, making it difficult to form close interpersonal relationships (8), let alone generating lasting and stable emotional support. Social media, however, can break the limitations of time and space and provide a technological platform for long-term patient support. In particular, social media can be used as an extension of offline interpersonal relationships and is proven to be effective in creating new connections and maintaining the original ones, thus providing an emotional bridge (56). Through social support groups, patients can share their feelings and difficulties with others who are going through similar experiences, and they are free to express their concerns without fear of burdening their social networks or incurring disapproval (57). In this way, social media is increasingly becoming a new way to build emotionally influential social networks for patients and to promote ongoing compliance with health-protective behaviors (7, 58).

### Patient Self-Efficacy and Disease Self-Management

Self-efficacy has been recognized as a mediator of health behaviors in that when people are ill, they will make the necessary efforts to cure their illnesses and continue with their lives in the future if they believe they can control and adhere to treatment, recovery behaviors, and healthy habits (59). Medical research has demonstrated that self-efficacy has an important influence on behavioral change. Increased levels of self-efficacy have a positive impact on health behaviors, enabling people to improve their health through the regulation of their attitudes, emotions, and behaviors toward health (60), thus facilitating symptom control and improving quality of life (61). Therefore, fostering a sense of high performance in patients is increasingly recognized as a goal of long-term care (62).

Four sources contribute to the construct of self-efficacy (59, 63): (1) past achievements and performance accomplishments, i.e., the ability of patients to perform desired behaviors and accomplish milestones, thereby creating a strong sense of efficacy to accomplish future rehabilitation tasks; (2) vicarious experience, i.e., observing other patients' successes and failures to imitate valuable rehabilitation behaviors; (3) verbal persuasion, i.e., verbal persuasion by health care providers, relatives, and other patients to convince patients of their ability to recover; and (4) emotional persuasion, which is an attempt to strengthen the patient's control over his or her physical and emotional state so that they can cope with the disease with a positive and optimistic attitude. The four aspects mentioned above have been used as the basis for clinical pathway interventions developed by hospitals to train patients in self-efficacy, usually through text-based education, provision of an interactive environment for patients, and training family members (64).

In the field of medical therapy, cancer-related self-efficacy refers primarily to the act of self-control and participation in treatment (65). Correspondingly, the Chronic Disease Self-Management Program (CDSMP) was introduced in the medical community to emphasize the central role of the patient in managing the disease (66). As most cancer survivors require ongoing medication, their diet, hydration, exercise, mood management, leisure activities, and social life all need to be adjusted depending on their disease status (12). Therefore, it is important that they have the confidence and skills to manage their long-term health. Effective self-management has the beneficial outcome of ultimately reducing the recurrence of disease and the financial burden of disease (67). Thus, it can be deduced that self-efficacy has an important impact on patients' disease self-management behaviors. To improve self-management in the disease process, patients must improve their self-efficacy and believe that they can control their disease (7, 68). The higher the patient's self-efficacy, the higher their independent self-care ability (69). Although previous studies have confirmed that enhancing patients' self-efficacy is one of the most important ways to improve patient self-care, most of them focus on offline hospital care behaviors, and there is no study on the effect of media-based patient communities on the construct of self-efficacy (61). Therefore, this study investigates how WeChat groups contribute to cancer patients' sense of efficacy and enhance their self-management behaviors by referring to the four sources of self-efficacy mentioned above. How do these groups differ from offline sources of self-efficacy construction?

In summary, a practical summary of cancer patient care revealed instrumental social support and emotional social support as important factors in improving patients' cure rates and quality of life. From a patient's psychological perspective, the four aspects of disease self-efficacy are also considered to have a positive effect on patients' confidence in healing and participation in treatment. Moreover, the development of media devices and applications has provided a new platform for patient support model. Therefore, in this study, we used a mutual help WeChat group organized by cancer patients as a research field to investigate how social media can positively affect cancer patients in terms of instrumental social support, emotional social support, and disease self-efficacy. The research questions are as follows: (1) assess the differences in the role and impact of patient support WeChat groups, doctor-patient support, and offline support/web community support (2) explore the effects of cancer patients' daily interactions in a WeChat group on their perceived instrumental and emotional social support (3) determine how the WeChat group affects patients' sense of efficacy. By investigating these aspects of WeChat support systems, this study aims to reduce the pressure of patient care in hospitals, provide a channel for patient communication and self-help that can effectively improve the treatment process and long-term quality of life, and enrich health communication theories and local experiences in China.

## Research Design

### Research Methodology

Netnography is the process of ethnography in a virtual environment on the internet, capturing complex online cultural and social phenomena through participatory observation and in-depth interviews (70). In order to overcome the limitations of the researcher in being an bystander, several scholars have strongly suggested that the researcher should participates in online observation and discussion in person, together with in-depth interviews, to obtain complete data (70–72). Through this method, not only can the researcher's questions be answered but the researcher's analytical views can also be examined, and their true meaning reconfirmed (73). Moreover, WeChat as a kind of ethnographic writing culture, which has both authenticity and self-reflexivity, can amplify and depict virtual social reality in-depth (74).

Therefore, in order to answer the research questions, we first observed the daily conversations of the patients in the group chat as members of the WeChat group, especially on the topics of pre-, mid-, and post-treatment information, treatment knowledge, care practices, and mental health, emotional, and interpersonal support, and examined their online activities to grasp the interactive practices of cancer patients and survivors in the WeChat group. We also conducted semi-structured interviews to complement the observed WeChat group interactions, understand how social media is used and interpreted by cancer patients, and explore how the WeChat group provides instrumental/emotional social support to patients. Additionally, we explored how WeChat group interactions contribute to patients' sense of efficacy and thus enhance their self-management behaviors, i.e., how beliefs contribute to behaviors, thus linking online interactions with their offline treatment and recovery processes. Questions asked such as: How often do you speak in the WeChat group? What is the topic of your daily WeChat group chats? What do you think is the difference between the WeChat group and the mutual aid organizations you have participated (if any) in in the past? Do you think that after joining the WeChat group, your confidence in curing diseases has increased? Do you think WeChat groups make you more active in treatment? …The data collection period for this study was 242 days from November 1, 2020, to June 30, 2021. In each interview, we first made an appointment with the respondent and negotiated the time of the online video interview. Each online video interview is conducted by the researcher and the respondent through the WeChat platform, and lasts about 2-h. Before the start of the interview, the researcher proactively informed the purpose of the interview, and signed an interview agreement and authorization for oral recording/transcription with the respondent. Prior to the formal interview, three respondents were invited to take a test to understand the suitability of the interview protocol and to increase the sensitivity of the researcher on this topic.

### Research Subjects

For this study, we selected the nasopharyngeal carcinoma WeChat patient support group “Victory in Distress” as the survey field. The reasons for using nasopharyngeal cancer as a representative sample in this study are as follows: (1) This disease type is representative as the Chinese population has become prone to nasopharyngeal cancer, accounting for 38.29% and 40.14% of the global incidence and mortality of nasopharyngeal cancer, with higher incidence and mortality rates than the world average [1.2/100,000 and 0.7/100,000, respectively; (75)]; (2) The treatment process is representative, i.e., the treatment process for nasopharyngeal cancer includes chemotherapy, radiotherapy, and surgery, which are necessary for most cancers, the total treatment period is about 3–6 months, and there are treatment side effects and long-term rehabilitation needs; (3) Patient participation in social media is operational, i.e., they are able to take care of themselves for most of the treatment process and their communication with other patients using mobile phone is not affected; (4) Long-term observation is facilitated, i.e., the prognosis of nasopharyngeal cancer is good, almost all patients can return to society and resume normal life after the first treatment, and the survival period is also several years (76).

The WeChat group was selected for this study for the following reasons: (1) The WeChat group was established in 2018, has been active for more than 3 years, and a total of 82,389 chat messages were posted in the group during the study period, with at least 20 group members posting more than 300 conversations per day, thus providing a rich source of information for the study; (2) There were 142 members in the group as of June 30, 2021, of which 129 were patients and 13 were family members—this does not exceed Dunbar's number (150), meaning that theoretically, all members of the group know each other and are aware of each other's relationships (77); (3) Membership in the group was strictly limited and patients could only apply for membership through the group leader, who asked each applicant about their illness and treatment status to ensure authenticity in advance. Additionally, each group member noted their screen name, treatment hospital, tumor stage; (4) The members of the group all had one-on-one relationships through offline acquaintanceship, and the group leader and members actively monitored whether salesmen or those who provided false identities were present—once identified, such individuals were immediately removed from the group; (5) The researcher initially joined the group as a family member, had good interactions with the patients, and gained their trust.

Regarding the in-depth semi-structured interview interviews, the researcher recruited respondents by identifying themselves, the research questions, and the purpose of the study in the WeChat group. Previous studies have suggested that personal background variables such as gender, marriage, work experience, and family socioeconomic status may affect self-efficacy. In this study, we sought to obtain diverse and rich data from the abovementioned WeChat group of nasopharyngeal cancer patients in person regardless of gender, age, income, or region. Recruitment of respondents until reaching information saturation, that is, when the coding of the last few respondents is repeated with the previous respondents, the data collection is stopped (78). In addition, to respect personal privacy and confidentiality, the names of the respondents were coded using their screen names (pseudonyms) and some sensitive information was blurred. Twenty two patients were identified, including 17 males and five females, of which eight were primary school graduates, nine were secondary school graduates, and five were university graduates. Most of them were aged 35–65 years. The disease duration ranged from 1 to 10 years, and tumor stages 1 to 4 were represented.

After 22 interviews, the interview data were collated into verbatim drafts and used thematic analysis for analysis. Also, in order to obtain objective results, three researchers were engaged to carry out the following process to reduce the rate of erratum by investigator triangulation (79). First, the researchers carefully found out the content related to the research topic in the verbatim manuscript, and formed a preliminary conceptualization based on the relevance of the topic and content. Second, the researchers coded the verbatim drafts, grouping the words/phrases/sentences into categories, searching for meaningful units in the material, and grouping them further into categories to further conceptualize the empirical data. Finally, the main axis of coding was carried out, i.e., after categorizing, comparing, and summarizing the themes of the abstracted content, the codes with the same attributes were regrouped to identify the recurring core categories in the data. Based on the research purpose, this research adopted open coding in the social support of WeChat groups, and constructs answers from respondents' responses. At the same time, a fixed core category is adopted in the self-efficacy of patients, that is, the four sources of self-efficacy (past achievements and performance accomplishments, vicarious experience, verbal persuasion, and emotional persuasion) are used as the core category.

## Research and Analysis

### As a Timely, Accessible, Ongoing, and Accurate Source of Experience and Knowledge

About half of cancer patients are disadvantaged in their ability to obtain, process, and understand cancer-related information and make decisions accordingly because they lack the necessary knowledge (80). Additionally, the patient may also be disturbed by the negative emotions of the treatment to understand and remember disease information. Therefore, it is important to give the patient linguistic, logical, and scriptural matching information when they need it (39). The most important daily interaction in the WeChat group was “answering questions for rookie patients.” Members of the group shared disease and treatment information in a repetitive, complementary, and extended manner in the group chats. In particular, the “one-question-one-answer” format was considered “simple” and “straightforward” by the patients interviewed, “until they understand.” When interviewed patients encountered problems related to disease and care, their “first reaction” was to ask questions in the group, and experienced patients would reply to the questions within a short period. This study found that WeChat groups are active, real-time chat rooms, and the biggest difference between them and web-based patient communities is the timeliness of the information received. On the one hand, web-based patient groups usually require a computer to log in, so patients cannot get information when they are in the hospital, in the ward, or outside or when they do not have a computer at home; on the other hand, the update rate of web-based patient groups is infrequent, and questions are often not answered for several days. Therefore, 90% of the patients surveyed said that the most important advantage of WeChat is that they get answers to their questions about treatment and care “quickly” and “almost within 3 min” when they ask for information. When discussing the most important functions and advantages of WeChat groups, some respondents stated:

The most convenient thing is that I can open my mobile phone and ask a question and someone will respond quickly. So now I'm used to asking questions whenever I don't understand something. Before I joined the group, if I didn't understand something, I had to look it up on the internet, and I couldn't find anything even after half a day… It's really convenient to have a WeChat group. (Interviewee: Lovely as you, male, 50 years old, diagnosed in 2020, mid-to-late stage).

In addition to the text-based interaction, WeChat's built-in multimedia and hyperlink functions allow for a more “intuitive,” “simple,” “beautiful,” and “interesting” presentation of previously complex and obscure disease information. The 15 respondents who have participated the website-based mutual help community all agreed that, compared to website communities, patients in the WeChat group can more actively learn, share, and receive disease-related information using hyperlinks such as web pages, public account, applets, and videos. Several primary and secondary school-educated respondents said that thanks to the easy-to-understand operation of WeChat, they can often access information with a simple click. Such an operating system not only overcomes the difficulties of using computers and reading books for patients with low education levels and in remote areas but also facilitates the development of patients' media literacy and their ability to search for and distinguish relevant information on the internet, hence providing a double benefit.

Additionally, our research showed, the discussion in the WeChat group extends from the disease itself to the daily life experiences surrounding the disease, which is difficult to achieve through traditional doctor-patient communication or web-based information sources and communities (81–84). Apart from the disease itself, cancer patients also have to deal with many other problems arising from the disease. These problems are not the responsibility of hospitals, some respondents also said, personal relationships do not provide practical advice because the individuals are not experienced, and web-based patient communities are not used because timely responses are not given. However, for all respondents, these everyday issues are just as important as information about the disease and even need to be considered a part of the cancer treatment. This dilemma is effectively solved by the patient support WeChat group, which brings together cancer patients with common life experiences to share these common experiences, from treatment to life, and accompany each other throughout the process. For example, in the early stage of diagnosis, respondents ask about the list of authoritative hospitals and doctors or preliminaries such as registration and hospitalization procedures; during the treatment process, they discuss their experiences in renting an apartment, diet structure and exercise routine, daily self-care steps, and even dealing with their work relationships as the main topic. The above information was very difficult to obtain in “books,” “hospitals,” and “websites.”

I kind of use the internet for my work, but I find it hard to tell directly on the internet, for example, you can see which doctor has a high ranking, but you can't see which doctor has a good attitude toward the patient. I did feel that I didn't want to go and be left out by the doctors when I was so sick. So I found a doctor who treats his patients like a spring breeze by asking everyone (in the WeChat group). (Respondent: Jack, male, 38 years old, diagnosed in 2019, mid-stage).
It's a good thing I found our group, it really helped me at that time, otherwise, I really didn't know where to rent an apartment in Zhong (Shan) Oncology Hospital when I had my radiotherapy… I couldn't find it online, and the book? It's impossible to tell you, right? (Interviewee: Sheng Xia, female, 35 years old, diagnosed in 2016, early stage).

WeChat groups also have an advantage over web-based patient communities in dealing with misinformation and disinformation. There are a large number of advertisements and unfiltered medical information on the Internet (43). Fifteen respondents with web-based patient communities experience all said that they do not know how to distinguish right from wrong when they see this information in the online community. Also, more than half of the respondents stated that they had been misled by misinformation. However, based on the interactive mode, WeChat groups can exist for a long time, and through their stability, can accumulate accurate knowledge and experiences. The concepts and information flowing in the group are also repeatedly screened, verified, and passed on from generation to generation through the test of time. The knowledge and experiences will rotate as old patients learn and newbies join. Most of the respondents said that through daily sharing and discussion, they were able to go from “knowing nothing” when they first joined the group to having a relatively complete understanding of the treatment process and being psychologically prepared. Later, when new patients join the group, the knowledge will be passed on further. According to the group leader, most of the patients who have been in the group long enough can learn from the experiences of other patients and combine them with their own conditions to tailor their care plans. For example, Chinese patients are often tempted to try Chinese medicine, or even believe that it is the key to a cure when facing cancer. One respondent said that when she was first diagnosed, a relative referred her to an “old Chinese medicine practitioner” who claimed that the cancer would disappear without the pain of chemotherapy and would never recur and that it was the immediate advice of the patient's WeChat group that “saved her life.” The group's accumulated experience in healing warned members to use Western radiation and chemotherapy for treatment. However, they were also told that they could choose to use Chinese herbal medicine to alleviate the side effects of treatment and long-term conditioning. Through this exchange and sharing, some tips on the daily use of Chinese herbs were also established. On this topic, the group kept reminding participants of the rational mindset of “not to be blind,” “to see a famous doctor,” “to pay attention to the usage and dosage,” and “to follow the advice of the attending doctor” when taking Chinese medicine.

From the daily interactions in the above group, it can be seen that discussions on health and rehabilitation knowledge and even sharing of relevant life experiences are the main topics. WeChat groups have become the main or even the only, source of information for most of the patients. Due to the chat room function on mobile phones, its real-time Q&A and multimedia features have compensated for the time and space constraints as well as the barriers of illogical language and emotional acceptance that prevent patients from fully communicating with their doctors. The long-term existence of WeChat groups has also enabled the improvement, continuation, and development of allopathic information. With increased time in the group, most of the patients who actively participate in the group can learn the corresponding disease knowledge and realize improvements in health and media literacy.

### As a Source of Emotional Support for the “Reality”

The confirmation of a cancer diagnosis is often devastating and causes a variety of negative emotions such as anxiety, fear, anger, shame, and loss of values (85). Physiological disorders such as insomnia, poor concentration, loss of appetite, increased sedation, and alcohol abuse as well as suicidal ideation may also occur (86). In addition, the nasopharyngeal malignancy is unique and the complications associated with treatment are unbearable for the patient and contribute to the overall deterioration of quality of life. The treatment process produces uncomfortable symptoms such as dry mouth, skin ulcers, otitis media, swallowing difficulties, nausea, and regurgitation, resulting in a sense of inability to live a normal life, irritability and hesitation, and a tendency for cancer patients to avoid coping and give in Han et al. (87). Such emotions were also reflected in the respondents, as almost every patient interviewed was initially very anxious due to the tradition of “talking about cancer,” thinking that they were “dying soon.” Half of the respondents reported that they were not going to undergo treatment or had given up halfway through because of emotions such as “fear,” “scared,” “not knowing what to do,” and “helplessness.”

Unfortunately, hospitals and relationships are not meeting the emotional needs of cancer patients as they go through a “life-changing” experience. On the one hand, doctors and nurses are overburdened with daily work and have no time to calm patients' anxieties. When asked if they had received any emotional comfort from their doctors, 80% of the respondents recalled “almost none.” Patients reported that each visit to the doctor took “ <3 min” and that “the attending doctor only gave a quick and routine explanation of the examination,” “I had no chance to ask questions, let alone take care of my emotions,” and “seeing that there were people queuing behind me, I was too embarrassed to say anything more and left after the examination.” On the other hand, a person is usually able to receive or experience moral support from others in the social network they have established [e.g., friends, colleagues, neighbors, family, and relatives; (67)]. However, most respondents reported that relatives and friends were not able to empathize with them because they did not have the psychological knowledge of caregiving and did not have shared life experiences.

My wife cooked me three meals a day but I was having chemotherapy and couldn't eat at all, and I was seasick for about a week but she would force me to eat all the meals. I knew it was for my own good but she really didn't understand the physical pain I was going through. I don't know how to tell her, and I don't think she understands me, so I end up arguing a lot. (Respondent: I love my family, male, 56 years old, diagnosed in 2018, early stage).

After cautiously turning to their old social networks and feeling unappreciated, patients turn to unfamiliar friends in the group for support and voice their feelings to each other. At this time, the reassurance of friends in WeChat groups is often helpful in improving emotions, relieving symptoms, managing pain, and maintaining a positive attitude. Many respondents reported that they had experienced full emotional support in the group, from being “new to cancer” to “living with cancer.” At the same time, social identity also comes into play, whereby clear identity influences the implementation of health behaviors advocated by the group, thereby facilitating health behaviors and treatment adherence (6). In other words, when patients are more involved in a WeChat group, they identify more strongly with their group and experience a greater sense of inclusion and belonging, which in turn leads to their own group-initiated awareness and behavior. At this point, it is even easier for group members than family member to persuade the patient to accept treatment and induce rehabilitative behaviors.

They all told me that nasopharyngeal cancer is also called “happy cancer” and that if you have to get cancer once, you are lucky! I see that they are all alive and well, so it gives me the confidence to fight through it! I believe I'll be a good man again in three months! (Interviewee: Yes, male, aged 47, diagnosed in 2021, mid-stage).

At the same time, online interpersonal relationships can also protect patients from the pressure to express their emotions publicly compared to real social capital (47). The public generally believes that nasopharyngeal cancer is caused by one's smoking behavior, thus condemning, rejecting, and even discriminating against nasopharyngeal cancer patients, who themselves may also develop feelings of guilt, self-blame, and stigma (50). Higher levels of stigma can result in a fear of society and people (17), i.e., negative emotional experiences in which patients fear rejection and blame from the outside world. After months of treatment, patients need to face one last emotional hurdle—social reintegration. It is at this point that the patient's sensitivity to social discrimination is most pronounced and there is nowhere to go. Once again, the WeChat group plays a positive role as an important force in boosting patients' confidence in facing society and reducing their sense of stigma. Ninety percentage respondents felt that their self-esteem had been damaged to varying degrees due to the changes in their appearance caused by the treatment, such as “losing weight,” “losing hair,” “having skin ulcers on the neck,” “having a pale complexion,” worrying that “bosses,” “colleagues,” and “neighbors” would look at them differently, and “not knowing how to face it.” The patients who had already returned to society gave courage and strength by recounting their experiences. After several months of interaction, the “cheering” and “encouragement” from these virtual relationships have become “a real spiritual force” in the eyes of the respondents.

Online emotional support alone is also enough to influence one's health status (51), and WeChat groups are effective in soothing patients' confusion at diagnosis, agitation at treatment, and anxiety at reintegration. Such emotional support also does have a significant positive effect on patients' quality of life. Emotional comfort clearly alleviates the patient's stress, helps to maintain a positive emotional experience and physical and psychological condition, and thus improves the ability to cope with the disease (88). In this regard, WeChat patient groups not only break time and space boundaries but also protect patients from interpersonal pressure, provide a mutually comforting exchange of common life experiences when the original social support structure is missing, and thereby providing patients with a brighter and more promising vision of the future in addition to practical solutions.

### The Role of Beliefs in Behavior

Although sense of efficacy is an internal self-assessment, it is largely influenced by external factors (89). Moreover, self-efficacy is considered to underlie behavioral changes in social cognitive theory (90), making the concept an important factor in predicting healthy behavior.

In this section, the four sources of self-efficacy constructs are explored to determine whether WeChat groups have a positive effect on cancer patients' health self-management behaviors through the communication of positive beliefs.

First, the past successful experiences of the cured patients had a motivating effect on the patients in the group. Eighty percentage respondents said that when they were first diagnosed, they “did not think they could be cured” and this thought also led to negative behaviors such as “not wanting to cooperate with doctors” and “not being motivated to go to the clinic” during the treatment process. Later, all respondent saw in a WeChat group that some people who had been through the treatment had completed it and returned to society, so they started to believe that they could “get through it.” Then, they actively engaged in treatment. Even after the treatment is over, the patient still has to face the sequelae, such as short-term taste disorder, skin redness and peeling, dry mouth, and long-term otitis media, radioactive molars, and skin fibrosis on the head and neck. All these “unpredictable bombs” make patients worry a lot about their quality of life and induce a constant state of “fear that the sequelae will come soon.” At this point, experienced patients can use their personal experiences to reduce their uncertainty and gradually offset their anxiety. These past experiences have helped patients to understand that daily practices such as “washing the nose,” “cleaning the teeth,” and “functional exercises” are effective in reducing the onset of sequelae and motivating them to adhere to their subsequent recovery behavior.

I might have to stay in rehab for the next 5 or 10 years. I thought it would be too hard to keep going. But a patient who has been cancer-free for 10 years still reminds us to do functional exercises regularly in the group every day, so when I saw how well he is doing, I insisted on following him. (Interviewee: Ah Suet, female, 61 years old, diagnosed in 2021, advanced stage).

Second, the patients in the WeChat group also became the targets of imitation, providing a behavioral basis for treatment and recovery. Apart from the negative emotions mentioned above, a more important reason for not implementing good self-management behaviors was that respondents did not know what to do. To summarize the reasons cited by the respondents, most of them are related to the following: (1) Due to a limited medical background and the low doctor-patient ratio, oncology staff are already overwhelmed with the basic treatment tools, cannot afford to provide more care knowledge, and “only emphasize what they have to do” (60% respondents); (2) Worse still, when patients leave the hospital, they have no place to get “practical information,” especially on the “smallest daily acts,” such as “How should I make five red soup?” or “How to make the hair that fell out after chemotherapy grow back thicker?” (70% respondents) In this regard, the WeChat group consists of people who are interested in the “small daily behaviors,” such as how to cook five red soup for blood. In this case, the “old bird patients” in the WeChat group became good targets for imitation. Nearly all respondents admitted that they were imitating the behavior of experienced patients in the WeChat group. By observing the behaviors of patients similar to themselves, the respondents were able to acquire self-care and self-efficacy management experiences (91) on the one hand and increase their sense of control mentally (92) on the other, which led to more positive recovery behaviors. “I owe my good health to the nutrition they taught me, especially the recipes I get from my sister every day.” (Interviewee: Lai Wei, male, 49 years old, diagnosed in 2020, early stage).

Again, verbal persuasion became an important source of motivation. As mentioned in the previous section, probably due to the traditional Chinese “face” culture, most patients do not have the courage to seek support from their original social network due to the stigma associated with the illness (50). Instead, patient groups form new collectives in which patients gain social/group identity, a conceptual shift from “I” to “we” (93). Thus, when the individual respondent belongs to a similar social group, they are connected to other members and provided with self-esteem, social support, a sense of belonging, wellbeing, and hope from building collective beliefs, which leads to common goals (94). In addition, the give and take of each member in the group are balanced. According to the group interaction model, each patient can become a sharer rather than a mere taker. Whether sharing health knowledge, life experiences, or even simple words of encouragement, At least more than half of the respondents feel that they are “contributing to the group.” This can be interpreted as a process of empowerment (95). Giving help to other patients also made the respondent feel more empowered to gain control, thus increasing their self-confidence and positive behavior. At this point, encouragement and discipline from other members of the group also had a multiplier effect on the patient's behavioral self-management.

I think it's amazing that after being in the group for a long time, we feel like we are fighting side by side to defeat the devil of cancer. So when we say words of encouragement to each other, I feel that they are sincere, so I will be motivated to keep going. When I don't want to go on, it's better to listen to the advice of my patients rather than anyone else. (Interviewee: Heart, male, 35 years old, diagnosed in 2020, advanced stage).

Finally, the role of emotions should not be overlooked. Negative emotions can weaken patients' compliance with medical treatment, leading to poorer outcomes and seriously reducing the quality of life of patients and their families (60). Enhanced psychological interventions can help reduce emotional distress and enhance the patient's resilience to “dancing with cancer” (96), which may also improve the patient's quality of life and reduce symptoms (97). However, treatment facilities do not provide adequate consultation services due to a lack of staff and funding (54). In addition, physicians are often not trained in professional communication skills, which may lead them to avoid emotional interactions with patients. In line with the analysis in the previous section, it was concluded that WeChat groups as a source of emotional support do in fact provide a positive emotional stimulus to patients. All respondents concluded that emotional support from a group of friends could help to reduce unpleasant emotions and make them more likely to maintain positive emotions when coping with physical discomfort and depression.

I went to Baidu to look up information about nasopharyngeal carcinoma, and it was so scary that I couldn't sleep all night. But then I joined the group and we encouraged each other not to think about it, to be active in treatment every day, and to sleep at night. It really calmed me down and encouraged me to accept the treatment more positively. (Interviewee: Sun Miracle, female, 38 years old, diagnosed in 2019, mid-to-late stage).

Taken together, cancer patients' sense of efficacy is a key influencing factor in their treatment and prognosis outcomes and has been recognized as an important predictor of patient recovery (98). WeChat groups do increase the sense of efficacy in the four ways mentioned above, and higher levels of self-efficacy have been confirmed by many studies to promote patients' self-management and reinforce healthy behaviors (12, 60). Thus, patient support WeChat groups do play an obvious role in cancer patients' treatment and future recovery behaviors.

## Discussion and Conclusion

Regular use of the internet can synthetically improve the wellbeing of cancer patients (99). The internet and smartphones are also shown to promote behavioral changes for recovery (7). This study found that the patient mutual aid WeChat group as a social media provides both instrumental and emotional social support which can help improve the patients' sense of control over their disease, thereby generating a sense of efficacy in fighting the disease and increasing awareness of actively participating in the treatment. In this regard, this study found that the positive effects of the WeChat group are mainly manifested in the following aspects:

First, when compared with web-based mutual aid communities, the WeChat group is a more timely, popular, continuous, and easier to distinguish correct information and plays a more positive role in the rehabilitation of patients. In terms of instrumental social support, the importance of disease information for patients cannot be overstated. The virtual nature of the network society gives people more freedom and space for expression. Those with low health and media literacy are likely to be overwhelmed by complex professional health information that may easily be misinterpreted or misrepresented. In this situation, information-searching can result in significant psychological or emotional stress, leaving patients unsure of how to follow medical recommendations and discouraged from further attempts to seek them out. The emergence of WeChat patient groups addresses such cognitive obstacles by bringing together people with common life experiences and similar informational needs in an environment free of commercial influences on information availability. The information shared in a mutual aid group chat is more likely to be relevant and accessible to other members of the group. Because members have similar diagnoses, treatment experiences, and mental histories, the communication and interaction within the group is simple, direct, and timely, and patients are able provide spiritual and emotional comfort to one another out of empathy as well as based on their own treatment experiences and results. When shared with the group, complicated medical information can be parsed collectively or by more experienced members, and members can also make timely corrections to improper or erroneous health information, thereby promoting the dissemination of accurate and necessary health information.

Second, WeChat groups provide a community based on a shared understanding of needs and challenges. Past research on social support in medical contexts has focused on patients' families and friends to provide understanding, care, companionship, and helping patients confront and manage patients' negative emotions (50), but the findings of this study may appear to contradict the assumptions based on such research. We found that while patients may avoid society out of fear of stigma—that is, fear of encountering rejection and blame—they may also be uncomfortable with the excessive care shown to them by relatives and friends. The value of WeChat groups is listening and understanding based on common life experience and emotional disturbances. Other patients in the group are more likely than healthy friends and relatives to have the knowledge and empathy necessary to listen and respond appropriately to the worries of their peers, thereby creating an outlet for patients to vent the emotions and stresses that they may feel unable to share with their friends and family, and serving as a source of social recognition and encouragement. Through honest communications and interactions within the group, patients can form a healthy, stable community and maintain a sense of social belonging. Maudgal (100) came to similar conclusions through a study on patient support groups on WhatsApp. The friendships established in these virtual spaces contribute to real support networks both by increasing the availability of relevant information and by providing advice and compassion based on shared experiences.

Combined with China's medical environment, the patient WeChat group may also bridge the urban-rural gap with regard to the uneven distribution of medical resources. At present, the level of hospitals and clinics in various cities in China is uneven, and there is no shortage of cancer patients who suffer from substandard treatment plans, incomplete medical advice, or even misdiagnosis. Patients attending hospitals in remote areas often have low health literacy and are most likely to be a disadvantaged group lacking in education and socioeconomic power; therefore, their condition is likely to remain unhealed or even aggravated by their lack of understanding and knowledge thereof (101). WeChat groups are an important source of information with almost no professional gatekeeping and are easily accessible even to patients with low health literacy in remote areas. Patients who would otherwise have very limited access to comprehensible information about their condition can obtain it quickly and easily from their peers in the group, and group members may also be able to recommend diagnosis and treatment services based on cost, results, and quality of care. In cases where patients are unable to undergo diagnosis and treatment, the group may provide alternate sources of support. Patients experiencing financial difficulty may also request donations from the group, whose members pass the request on to their own social networks in order to broaden the support base.

In addition, from the perspective of self-efficacy, the research results showed that for most patients, WeChat groups can increase their confidence in healing and actually promote the implementation of patient self-management behaviors. The results may extend the empirical research experience of disease self-efficacy from offline to online. In the past, many studies have proved that the support services provided by hospitals and charity organizations can increase the patient's self-efficacy, thereby increasing the overall probability of disease recovery (7, 102–104). However, due to the limitations of time, space and manpower, the service cannot be popularized offline. From the results of this study, when almost patients know that they had cancer, they were initially prone to depression, pessimism, despair, and other such negative states and behaviors due to lack of understanding of the disease. However, if we examine from the four sources of self-efficacy construction summarized by Bandura (59), we found that the daily interaction experience of WeChat groups does have a positive effect on: learning from past successful care experiences, imitating the behavior of patients in the latter stage of rehabilitation, engaging in verbal persuasion and encouragement, and receiving emotional support. And research showed when patients indicated that they have increased confidence in fighting cancer, they also obviously reflected this inner strength in their behaviors, that is, they are more active in treatment, more coordinating with daily rehabilitation activities and maintaining good living habits. This proves that the positive effect of disease self-efficacy on health self-management behaviors (12, 60) also exists in the context of the Internet.

We suggest that future researchers build on the findings of this study in the following ways: (1) This study found that patients with the same disease spontaneously formed a WeChat support group which quickly proved conducive to alleviating psychological and emotional problems, such as isolation and overwhelm, perceived by patients. The concept of social identity (105) may be applied to investigating the underlying mechanism of this phenomenon, as members of such WeChat groups construct a strong and clearly defined group identity through interaction, and the specific health behaviors advocated by members of a group are more persuasive than those advocated by outsiders. (2) From the perspective of health risk communication, risk perception is usually based on emotions, trust, and personal or relative experience ((106), and WeChat groups fit this logic. Perhaps, in the future, this theory can be used to explore whether WeChat mutual aid groups are more effective than traditional doctor-patient mutual help and web medical consultation in dealing with the lack of health information, navigating disease risks knowledge and emotional stress that have positive moderating and guiding effects. Overcoming cognitive barriers through sharing knowledge, reducing emotional pressure through a sense of efficacy, and self-management behavior all have a positive impact on risk communication and response. (3) Self-efficacy beliefs were found to be significantly and positively correlated with social support from peers (107). And helping chronic diseases patients to build an effective social support system can improve their self-confidence and self-management (108). In terms of the four dimensions of self-efficacy explored in this study, successful experience in curing patients and imitating patients' behavior may also fall under of instrumental social support, verbal persuasion and emotional support may also fall under of emotional social support. Therefore, in the future, the relationship between social support and self-efficacy may be explored to examine whether social support has a positive relationship with disease self-efficacy. (4) Some studies have indicated that self-management behavior may be counterproductive to self-efficacy (16). Because one of the identified sources of information about self-efficacy is past success experience, patients who implement effective disease self-management may also may feel more confident in achieve remission. In summary, future research may be able to more thoroughly investigate effective health communication and disease management in contemporary media environment from a variety of perspectives, including those mentioned above, and provide further theoretical basis and practical references for medical care practice.

## Data Availability Statement

The original contributions presented in the study are included in the article/supplementary material, further inquiries can be directed to the corresponding author/s.

## Ethics Statement

Ethical review and approval was not required for the study on human participants in accordance with the local legislation and institutional requirements. Written informed consent to participate in this study was provided by the participants.

## Author Contributions

FZ was responsible for writing the initial draft and conducting the study. LP was involved in the whole study and manuscript revision. ZQ provided supplementary and modified materials. All authors contributed to the article and approved the submitted version.

## Conflict of Interest

The authors declare that the research was conducted in the absence of any commercial or financial relationships that could be construed as a potential conflict of interest.

## Publisher's Note

All claims expressed in this article are solely those of the authors and do not necessarily represent those of their affiliated organizations, or those of the publisher, the editors and the reviewers. Any product that may be evaluated in this article, or claim that may be made by its manufacturer, is not guaranteed or endorsed by the publisher.

## Footnotes

^1^This group was created without the incentive of health professionals and it does not have a health professional mediating it.
